# Pericallosal Lipoma and Cortical Dysplasia Masquerading as a Glioma

**DOI:** 10.7759/cureus.2728

**Published:** 2018-06-01

**Authors:** Brian L Anderson, Michael Sather, Jennifer Baccon, Krishnamoorthy Thamburaj

**Affiliations:** 1 Department of Neurosurgery, Penn State Milton S. Hershey Medical Center, Hershey, USA; 2 Pathology, Penn State Milton S. Hershey Medical Center, Hershey, USA; 3 Department of Neuroradiology, Penn State Milton S. Hershey Medical Center, Hershey, USA

**Keywords:** midline lipoma, epilepsy

## Abstract

Intracranial lipomas represent approximately 1% of intracranial lesions generally felt to represent the abnormal persistence of the meninx primitiva and are commonly accompanied by various developmental brain abnormalities. We report a case of midline intracranial lipoma and evolving frontal lobe fluid-attenuated inversion recovery (FLAIR) abnormality concerning for glial neoplasm in a patient with intractable epilepsy. Our case shows evolving magnetic resonance imaging (MRI) features over two decades raising suspicion for low-grade neoplasm which was ultimately found to represent cortical dysplasia.

## Introduction

Intracranial lipomas are rare lesions generally felt to represent the abnormal persistence of the meninx primitiva. They represent approximately 1% of intracranial lesion and are commonly accompanied by various developmental brain abnormalities. Most lipomas reported are located near the midline and are pericallosal in nature with agenesis of the corpus callosum commonly observed [1]. Cortical dysplasia is often found in patients with other intracranial pathology including lipomas, neoplasia, and vascular lesions. An association between cortical dysplasia and intracranial lipomas located near the cortical surface has been reported [2,3]. We report a case of midline intracranial lipoma and evolving frontal lobe fluid-attenuated inversion recovery (FLAIR) signal abnormality concerning for glial neoplasm found to represent cortical dysplasia in a patient with intractable epilepsy.

## Case presentation

**Figure 1:**
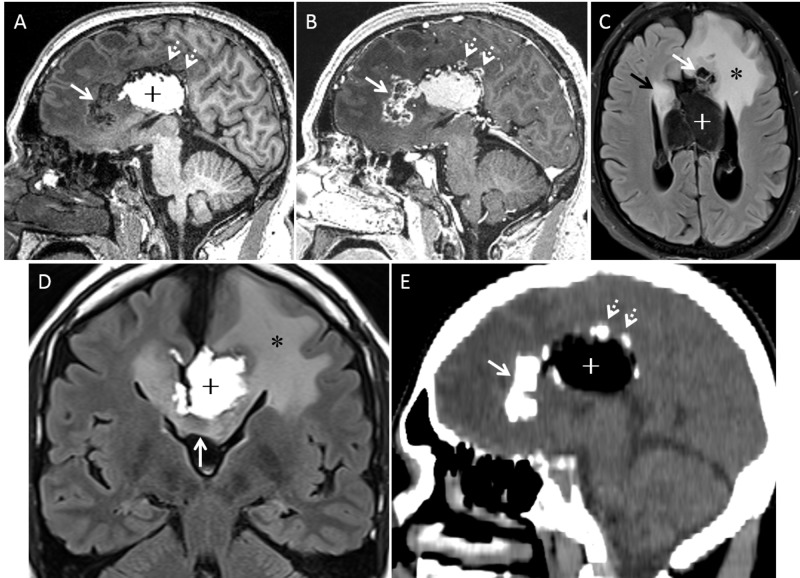
Preoperative images. A) Noncontrast left parasagittal 3D T1 image demonstrates nodular calcified lesion in the left anterior medial frontal lobe extending towards the cingulate gyrus (solid arrow). Small nodular calcifications (dotted arrows) at the junction of left cingulate gyrus and the pericallosal tubulonodular lipoma (+). B) Post contrast left parasagittal 3D T1 image shows peripheral enhancement of the nodular calcified lesions (solid and dotted arrows). C) Axial fat saturated FLAIR image demonstrates the nodular calcified lesion in the medial frontal lobe and cingulate gyrus (solid white arrow). Note the vasogenic edema involving much of the left frontal lobe (*). Lipoma appears dark from fat saturation (+). Focal edema in the right cingulate gyrus (black arrow). D) Coronal FLAIR image shows the lipoma (+) mainly located on the left side in the interhemispheric fissure. Genu of the corpus callosum shows extension of edema towards the right cingulate gyrus (arrow). Note the vasogenic edema left frontal lobe (*). E) Sagittal reformation of noncontrast CT demonstrates the nodular foci of calcifications (solid and dotted arrows) along with the hypoattenuating lipoma (+). CT: Computed tomography; FLAIR: Fluid-attenuated inversion recovery.

Our patient is a 43-year-old ambidextrous male with a 20-year history of intractable seizures. His seizure semiology typically included a hot flash and other sensory auras with evolution into focal motor activity predominately on the right. Workup for seizure etiology included magnetic resonance imaging (MRI) revealing the large midline lipoma, partial callosal agenesis, and an adjacent lesion in the left frontal lobe presumed to represent a glial neoplasm based on the radiographic appearance. The left anterior medial frontal lobe lesion consisted of nodular calcification with peripheral enhancement and extensive FLAIR signal changes involving bilateral cingulate gyri and the left frontal lobe (Figure 1).

Medical management efforts had failed to control his seizures and he was referred for surgical treatment options. The enhancing lesion was noted to progress with additional imaging and the extent of edema also expanded. The concern for progressive glial neoplasia and poor seizure control prompted a recommendation for surgical resection. The decision was made to proceed with neuronavigation-guided resection of the left frontal enhancing mass and partial frontal lobectomy utilizing intraoperative electroencephalogram (EEG), cortical mapping, and somatosensory evoked potentials (SSEP) monitoring.

**Figure 2:**
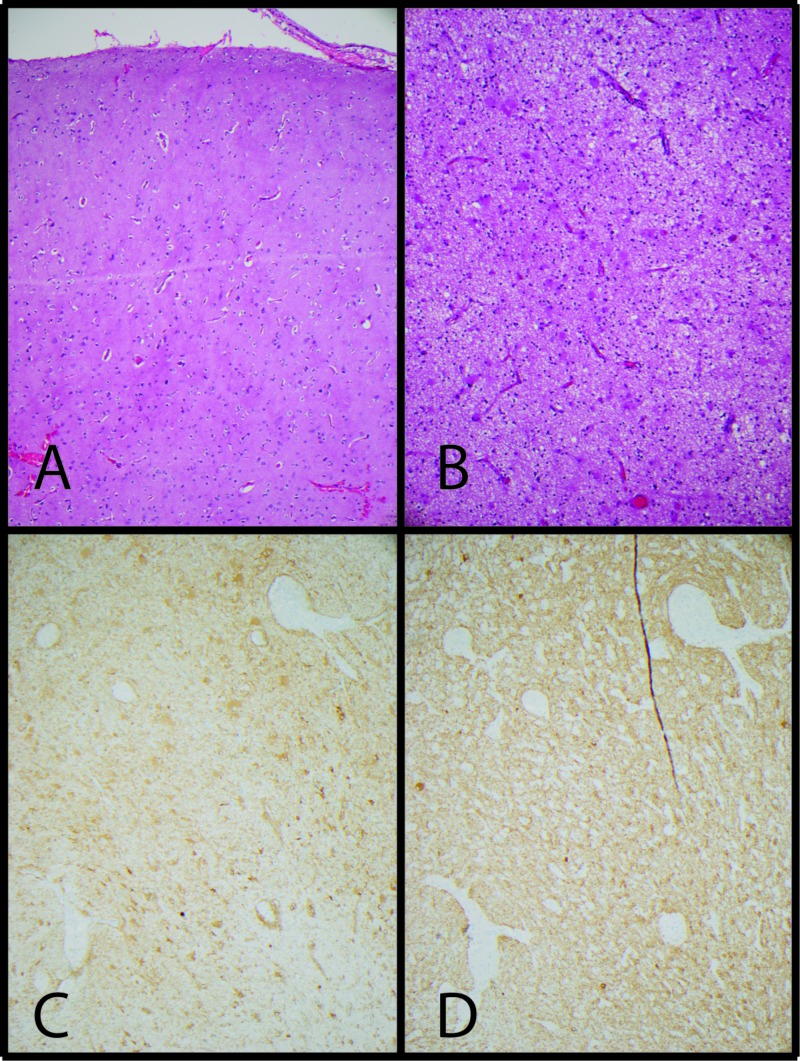
Focal cortical dysplasia, Palmini Type IA. A) The cortex, H&E 100x, shows mild architectural abnormalities and the white matter. B) H&E 100x shows associated gliosis. C) GFAP, 100x, immunohistochemical staining highlights the gliosis. D) NFP, 100x, does not highlight any dysmorphic neurons in areas of gliosis. These histologic features are diagnostic of focal cortical dysplasia, Palmini Type IA. H&E: Hematoxylin and eosin; GFAP: Glial fibrillary acidic protein; NFP: Neurofilament protein.

Intraoperative samples sent for frozen pathology were found to have Rosenthal fibers and focal calcification and felt to likely represent glial neoplasm. Permanent pathologic evaluation revealed a lipoma and focal cortical dysplasia, Palmini Type IA, in the adjacent brain. The mild cortical architectural abnormalities and associated white matter gliosis can be seen on hematoxylin and eosin (H&E) and glial fibrillary acidic protein (GFAP) stains and neurofilament protein (NFP) staining in the areas of gliosis shows no evidence of dysmorphic neurons, giant neurons or balloon cells (Figure 2).

**Figure 3:**
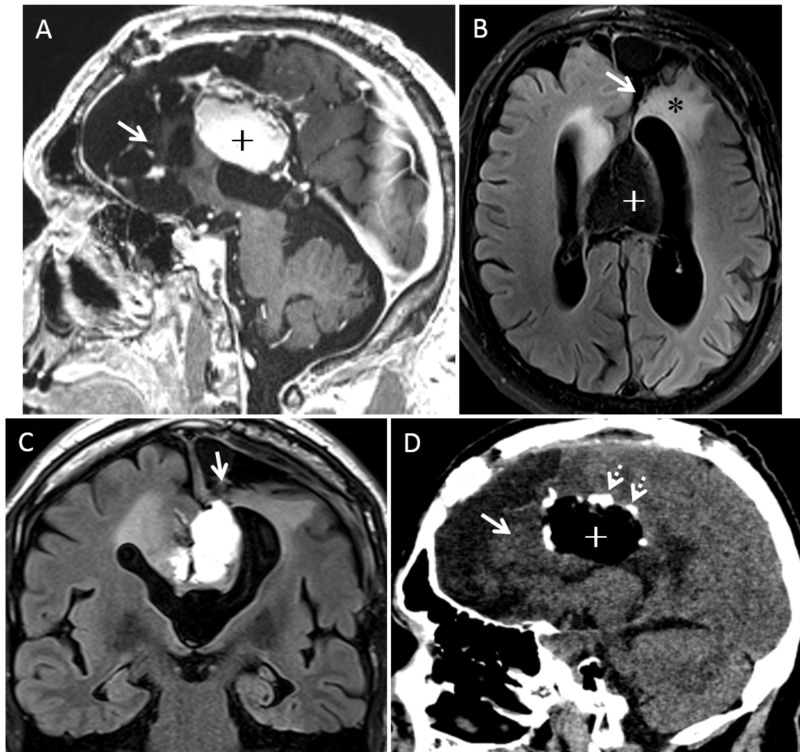
Post-operative images. A) Post contrast left parasagittal 3D T1 image shows resection of the large nodular calcified lesion in the left cingulate gyrus and medial frontal lobe (solid arrow). Pericallosal lipoma (+) is mostly intact. B and C) Axial fat-saturated and coronal nonfat-saturated FLAIR images show resection of the nodular calcified lesion and partial left frontal lobectomy (solid white arrow). Note the resolution of edema (*), mass effect and ex vacuo enlargement of the left frontal horn. Residual lipoma appears dark from fat saturation (+). D) Sagittal reformation of noncontrast CT demonstrates near total resection of the nodular calcified lesion in the left cingulate gyrus and frontal lobe. Residual calcifications are seen along the surface of the lipoma (+). CT: Computed tomography; FLAIR: Fluid-attenuated inversion recovery.

Post-operative imaging (Figure 3) shows a near-total resection of the peripherally enhancing lesion in the left frontal lobe and cingulate gyrus. A significant reduction in vasogenic edema was noted on FLAIR imaging. The midline lipoma was unchanged.

Post-resection recovery included a moderate supplementary motor area syndrome including hemiparesis, delayed speech, and changes in effect. These changes gradually resolved and the patient was found to be neurologically intact at outpatient follow-up. Seizure control occurred immediately after resection. Antiepileptic agents were continued and gradually removed over subsequent months without recurrence. The patient remained seizure-free three years post resection. 18-month follow-up imaging found stable T2/FLAIR changes with small unchanged areas of perilesional enhancement (Figure 3).

## Discussion

Intracranial lipomas are commonly accompanied by other abnormalities of development which are likely a result of a defect in differentiation of the meninx primitiva during neural tube flexion. Although various cortical abnormalities have been described in association with intracranial lipomas, the underlying pathology remains poorly understood [4,5]. A few sporadic reports are available describing cortical dysplasia and intracranial lipomas located most often at the convexity [5,6]. Neoplasia has been well documented to cause intractable epilepsy with several reports noting a significant coincidence of cortical dysplasia [7,8]. Our case shows evolving MRI features over two decades raising suspicion for neoplasm which ultimately were found to represent cortical dysplasia. It is unclear whether the intracranial lipoma and dysplasia arise together or if the presence of the lipoma resulted in cortical developmental abnormalities. The evolving MRI features were felt to indicate an active process of parenchymal change with neoplasm suspected.

## Conclusions

While concurrent abnormalities are often related to a common underlying developmental pathology, this is simply unknown. In the setting of lipomas, cortical dysplasias may change over time.
